# The Imd Pathway Is Involved in Antiviral Immune Responses in *Drosophila*

**DOI:** 10.1371/journal.pone.0007436

**Published:** 2009-10-15

**Authors:** Alexandre Costa, Eric Jan, Peter Sarnow, David Schneider

**Affiliations:** Department of Microbiology and Immunology, Stanford University, Stanford, California, United States of America; Institut Pasteur Korea, Republic of Korea

## Abstract

Cricket Paralysis virus (CrPV) is a member of the *Dicistroviridae* family of RNA viruses, which infect a broad range of insect hosts, including the fruit fly *Drosophila melanogaster*. *Drosophila* has emerged as an effective system for studying innate immunity because of its powerful genetic techniques and the high degree of gene and pathway conservation. Intra-abdominal injection of CrPV into adult flies causes a lethal infection that provides a robust assay for the identification of mutants with altered sensitivity to viral infection. To gain insight into the interactions between viruses and the innate immune system, we injected wild type flies with CrPV and observed that antimicrobial peptides (AMPs) were not induced and hemocytes were depleted in the course of infection. To investigate the contribution of conserved immune signaling pathways to antiviral innate immune responses, CrPV was injected into isogenic mutants of the Immune Deficiency (Imd) pathway, which resembles the mammalian Tumor Necrosis Factor Receptor (TNFR) pathway. Loss-of-function mutations in several Imd pathway genes displayed increased sensitivity to CrPV infection and higher CrPV loads. Our data show that antiviral innate immune responses in flies infected with CrPV depend upon hemocytes and signaling through the Imd pathway.

## Introduction

The fruit fly *Drosophila melanogaster* has emerged as a powerful organism for studying the basic principles of innate immunity due to the evolutionary conservation of innate immunity genes, pathways, and effector mechanisms as well as the ease with which the fly can be genetically manipulated. The *Drosophila* immune response includes the induction of antimicrobial peptides (AMPs), hemolymph coagulation, melanization, RNA interference (RNAi), and phagocytic insect “blood cells” called hemocytes. Genetic screens in *Drosophila* designed to identify mutants unable to induce humoral responses against bacterial and fungal infections uncovered two conserved signaling cascades, the Toll and Imd pathways, both of which lead to the activation of NF-κB transcription factors [1]. The discovery that the Toll pathway is required for the immune response in *Drosophila* [2], [3] led directly to the demonstration that vertebrate Toll-like receptors (TLRs) were also required for mammalian immunity [4].

The Imd pathway resembles the mammalian tumor necrosis factor receptor (TNFR) pathway [5] which plays a critical role in inflammatory responses and infectious diseases in humans, particularly viral infections [6]. In flies, the Imd pathway is activated by diaminopimelic acid (DAP)-type peptidoglycan present in the cell wall of Gram-negative bacteria and many Gram-positive bacteria [1]; loss-of-function mutants show increased sensitivity to a Gram-negative bacteria as well as a number of Gram-positive bacterial and fungal pathogens [7], [8], [9], [10]. The Imd pathway is activated from outside the cell through transmembrane pattern recognition receptors, namely peptidoglycan recognition proteins (PGRP)-LC and -LE [11], [12], [13]. The pathway can also be activated from the cytoplasm via a splicing variant of PGRP-LE [14]. Intracellular signaling is initiated through RHIM (RIP homotypic interaction motif)-like motifs found at the N-terminus of both PGRPs and the signal is relayed downstream via an unidentified adaptor protein [14]. PGRPs also interact directly with Imd, but this interaction does not seem to be required for signaling [14], [15]. Imd is located at a branch-point in the pathway downstream from PGRPs where the signal is transduced in two genetically distinct arms which converge again to drive the activation of the NF-κB transcription factor Relish (Rel). In one branch, the signal is propagated through dTAK1 (*Drosophila* transforming growth factor-activated kinase 1) [16], [17] to Kenny (Key) and Ird5 (*Drosophila* homologues of IKKγ and IKKβ, respectively) [18], [19] which promote phosphorylation of Rel. In the second branch, the signal is relayed via dFADD (*Drosophila* Fas-associated death domain protein) [20] to activate the caspase-8 homologue Dredd [5], [21] which is required for cleavage of phosphorylated Rel [5], [22]. Rel cleavage leads to its activation and translocation into the nucleus where it promotes transcription of AMP and other immune responsive genes [7], [23].

Though immune responses against bacteria and fungi have been well-studied in *Drosophila*, our understanding of antiviral innate immune responses remains limited [24]. Zambon et al. (2005) showed that the immune response against *Drosophila* X Virus (DXV) in whole flies requires the Toll pathway and appears to depend on cellular rather than humoral mechanisms. This is supported by the requirement for Toll signaling in the control of Dengue virus infection of *Aedes aegypti* mosquitoes [25]. Studies with Drosophila C Virus (DCV) in cultured S2 cells established that the conserved clathrin-mediated endocytic pathway is essential for both viral infection and pathogenesis [26]. Flies infected with DCV induce a set of genes distinct from those induced during bacterial infections and whose induction depends in part on the Jak-STAT pathway [27] and may require virus replication [28]. Comparative studies between different viruses [29] and distinct classes of microorganisms [30] have uncovered shared and unique aspects of the *Drosophila* antiviral response [10]. At the cellular level, the RNA interference pathway has been shown to be an important component of innate antiviral immunity in *Drosophila* [31], [32], [33], [34], [35]. In addition to its role in RNA interference, the evolutionarily conserved DExD/H-box helicase Dicer-2 has recently been shown to control an inducible antiviral response in the fat body [36].

Cricket Paralysis virus (CrPV) was originally isolated from field crickets [37] and later shown to possess one of the widest host ranges of any insect virus. Besides replicating in *Drosophila* (order: *Diptera*) [38], CrPV is capable of infecting several insect orders such as *Orthoptera*, *Hymenoptera*, *Lepidoptera*, and *Hemiptera* [39]. CrPV belongs to the *Dicistroviridae* family of RNA viruses which have been isolated from a range of invertebrate hosts [40]. In crickets, CrPV particles can be found in the cytoplasm of cells of the alimentary canal, epidermis, and nerve ganglia [41]. Particles are roughly spherical and non-enveloped, with a 30 nm-diameter and icosahedral symmetry (T = 3) [42]. Their genome consists of a single strand of positive-sense RNA which is readily translated by the cellular machinery upon infection. The ∼9 kb genome contains two non-overlapping open reading frames (ORFs) whose translation generate separate polyproteins that are proteolytically processed into several non-structural and at least three structural proteins.

Here we exploit the lethal phenotype induced by intra-abdominal injection of CrPV into adult *Drosophila* to characterize a genetically tractable *in vivo* host-virus model system and identify genes involved in innate antiviral responses. We tested several biological variables and chose a set of conditions that generate a robust and reproducible lethal phenotype. We found that infection of wild type flies under these conditions did not induce a humoral immune response (AMPs) and was associated with depletion of hemocytes. We also determined whether the Immune Deficiency (Imd) pathway was involved in the immune response against CrPV. Infection of loss-of-function mutations in the Imd pathway led to increased sensitivity to CrPV infection and higher CrPV loads, demonstrating a decrease in resistance to the virus.

## Results

### Characterization of CrPV infection in *Drosophila*

**Figure 1:**
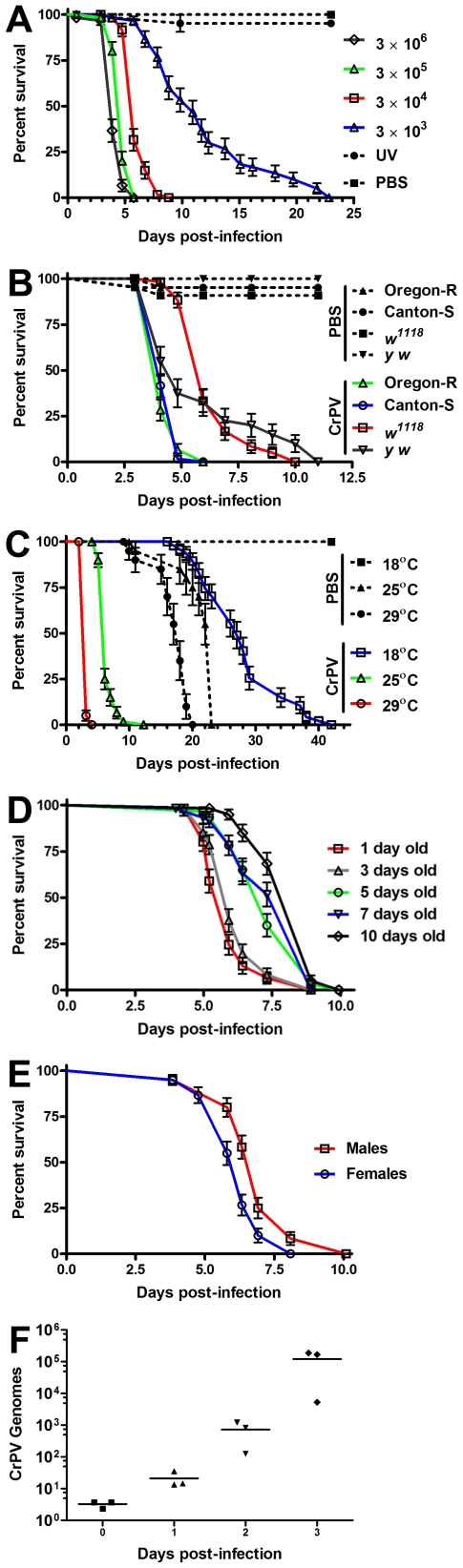
Characterization of CrPV infection in *Drosophila*. Three sets of 20 flies were injected intra-abdominally with CrPV and survival was monitored daily. (A) Dose-Response. Survival of 1-4 days old *w^1118^* male flies injected with 10-fold dilutions of a 3×10^6^ TCID_50_ CrPV suspension and incubated at 25°C. PBS and UV-inactivated CrPV were injected as negative controls. (p<0.0001, Logrank test for trend). (B) Genetic Background. Survival of 1–4 days old male flies of distinct genetic backgrounds injected with 3×10^4^ TCID_50_ CrPV and incubated at 25°C. (p<0.0001, Logrank). (C) Temperature. Survival of 3–4 days old *w^1118^* male flies injected with 3×10^4^ TCID_50_ CrPV and incubated at 18, 25 and 29°C. (p<0.0001, Logrank). (D) Age. Survival of *w^1118^* male flies spanning 1–10 days old injected with 3×10^4^ TCID_50_ CrPV and incubated at 25°C. (p<0.0001, Logrank test for trend). (E) Sex. Survival of 3–4 days old *w^1118^* male and female flies injected with 3×10^4^ TCID_50_ CrPV and incubated at 25°C. (p = 0.0002, Logrank). (F) CrPV titers. Five 1–4 days old *w^1118^* male flies injected with 3×10^4^ TCID_50_ CrPV and incubated at 25°C were homogenized at the indicated time points and the number of CrPV genomes determined by quantitative RT-PCR. Bars (A–E) represent mean values with standard error.

We found that injection of CrPV directly into the hemocoel (body cavity) of adult flies causes a lethal infection (Figure 1) as also reported by others [34], [43], [44]. Adult flies injected with 50 nl of a 3×10^8^ TCID_50_ (50% Tissue Culture Infective Dose) per ml suspension of CrPV (15,000 TCID_50_ CrPV per fly) in PBS die precipitously after 5 days post-infection (dpi). To ensure that death was caused by viral pathogenesis, we performed a dose response curve and used UV-inactivated CrPV and PBS as negative controls. As shown in Figure 1A, flies injected with UV-inactivated virus survived as long as uninjected or PBS-injected controls. In contrast, survival of flies injected with different doses of CrPV was inversely proportional to the amount of virions injected (Logrank test for trend, p<0.0001). To further characterize how other biological variables influenced the host's sensitivity to CrPV infection, we determined how genetic background, sex, age, and temperature affected the death kinetics of flies infected with CrPV. We observed that susceptibility to CrPV varies in different genetic backgrounds commonly used in *Drosophila* research (Figure 1B, p<0.0001) and that females are slightly more sensitive to CrPV infection than males (Figure 1E, p = 0.0002). We also found that susceptibility to viral infection is higher in younger adults compared to older adults (1–4 days old) (Figure 1D, Logrank test for trend, p<0.0001) and susceptibility is higher at higher temperatures (29°C>25°C>18°C) (Figure 1C, p<0.0001). These results allowed us to standardize the conditions in the experiments described below by using 1–4 days old *w^1118^* males injected with 50 nl of a 3×10^8^ TCID_50_/ml CrPV suspension and incubated at 25°C. We then used quantitative RT-PCR (qRT-PCR) to determine CrPV loads under these conditions. As shown in Figure 1F, CrPV loads increased exponentially in the days that precede fly death. These results indicate that the lethal infection caused by CrPV under these conditions provides a robust assay for the identification of mutations with altered sensitivity to viral infection.

### Humoral and cellular responses against CrPV

**Figure 2:**
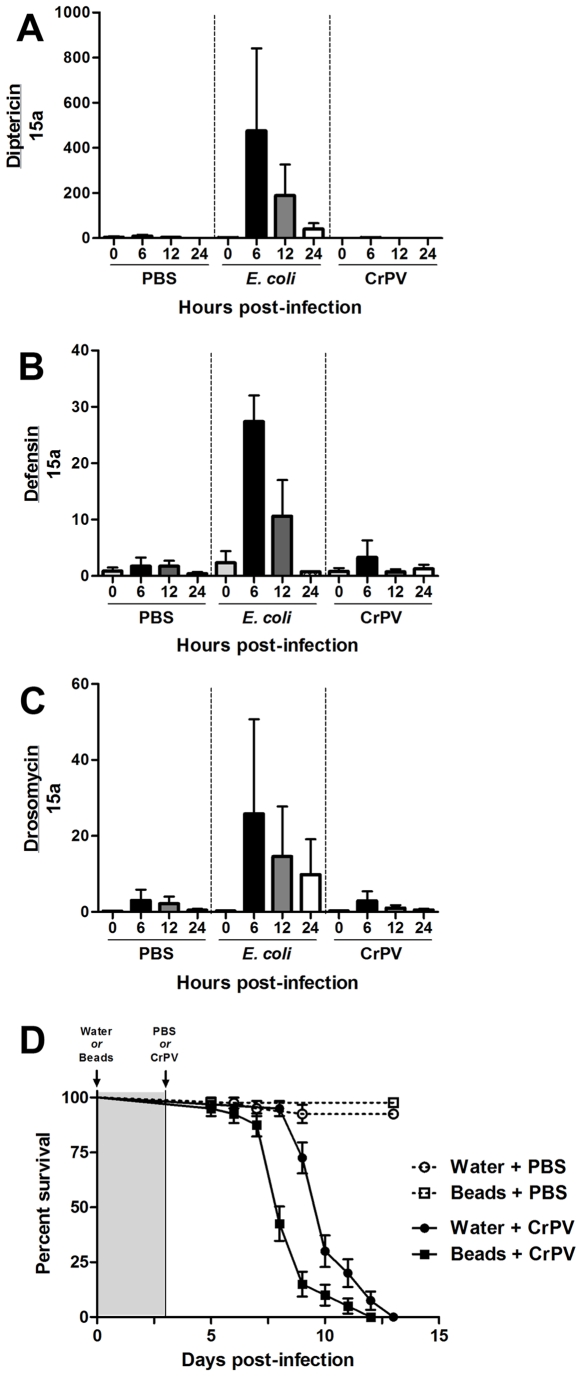
Humoral and cellular responses against CrPV. (A–C) AMPs are not induced by CrPV infection. Diptericin (A), Defensin (B), and Drosomycin (C) transcript levels in groups of five CrPV-infected flies were measured by quantitative RT-PCR at 6, 12 and 24 hours post-infection (hpi). AMP transcript concentrations were normalized to the levels of the ribosomal protein 15a transcript in each sample. PBS and *E. coli* were injected as negative and positive controls, respectively. Bars represent mean values with standard error. (D) Phagocytosis is involved in the immune response against CrPV. Three sets of twenty 1–4 days old males were injected intra-abdominally on day 0 with water (circles) or polystyrene beads (squares) to inhibit phagocytosis. Three days later, flies were injected with PBS (open symbols) or 3×10^4^ TCID_50_ CrPV (solid symbols) and survival monitored daily. Bars represent mean values with standard error. CrPV-infected flies die significantly faster when phagocytosis is inhibited (p<0.0001, Log-rank test).

To determine how the innate immune system responds to a CrPV challenge, we measured humoral and cellular immune responses to viral infection. The humoral immune response in the fly involves the rapid transcriptional activation of AMP genes in the fat body, followed by secretion of the peptides into the hemolymph. The induction of AMP genes depends upon signaling through the Toll and/or Imd pathway and therefore AMP expression has been used to monitor activation of these pathways during infection. For instance, Diptericin is largely induced by Imd signaling and Drosomycin induction is mostly driven by Toll signaling whereas Defensin is induced through activation of both Toll and Imd pathways. To determine if AMPs were induced during CrPV infection, we measured the transcript levels of several AMPs at 6, 12, and 24 hpi using qRT-PCR. Infection with *Escherichia coli* was used as a positive control because it can induce all of these AMPs. PBS (or LB medium, data not shown) injection was used as a negative control for baseline levels of AMP expression following wounding. Figure 2A–C shows that Diptericin, Defensin and Drosomycin were not induced during CrPV infection above background levels. Similar results were observed for other AMPs: Attacin, Cecropin, Drosocin, and Metchnikowin (data not shown). These results indicate that AMPs do not play a role in the immune response against CrPV under our experimental conditions. Moreover, if we relied solely on the use of AMP induction as a read out for Imd and Toll signaling, we would have come to the incorrect conclusion that neither Toll nor the Imd pathway is involved in antiviral responses.

The cellular immune response utilizes circulating cells called hemocytes that phagocytose, encapsulate, and kill invading parasites. To establish whether hemocytes play a role in innate antiviral responses, phagocytosis was permanently blocked in adult hemocytes by injection of 0.2 µm diameter polystyrene beads as described previously [21], [45]. Flies were injected with beads 3 days prior to CrPV infection and their survival was monitored daily. Flies injected with water prior to CrPV infection and flies injected with PBS after bead injection were used as controls. If phagocytosis by hemocytes was required during antiviral responses, flies whose hemocytes have been saturated with beads would display increased susceptibility to CrPV infection compared to controls. As shown in Figure 2D, flies injected with beads succumbed earlier to CrPV infection when compared to controls (p<0.0001). This result indicates that hemocytes are involved in the response against CrPV infection.

### Hemocytes are depleted during CrPV infection

**Figure 3:**
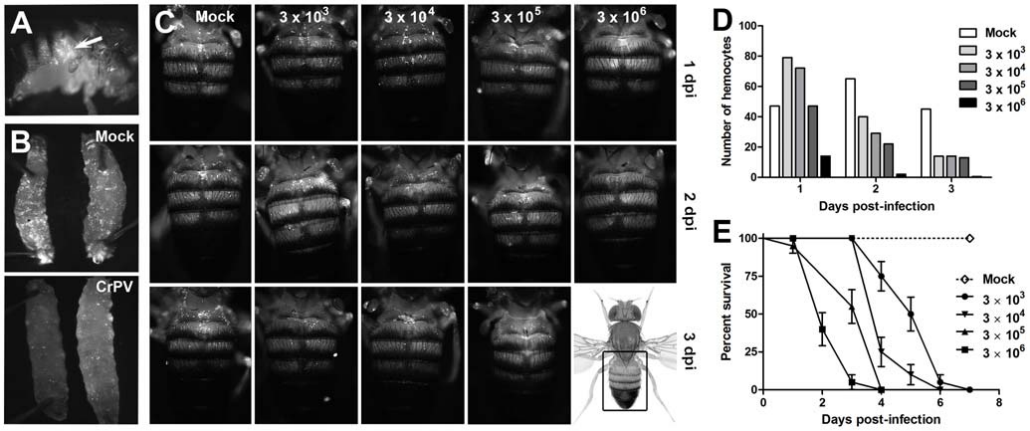
Hemocytes are depleted in the course of CrPV infection. Fluorescence microscopy of hemocytes in adult males (A and C) and larvae (B) infected with CrPV. Hemocytes were labeled with GFP whose expression was under the control of the Hemolectin (Hml) marker [72]. (A) Lateral view of 1–4 day old male fly infected with 50 nl of 3×10^6^ TCID_50_ CrPV and examined at 12 hpi. The arrow indicates the approximate injection site at the dorso-anterior abdomen. Note that hemocytes concentrate at the injection site. (B) Third instar larvae were injected with 50 nl of 3×10^6^ TCID_50_ CrPV and hemocytes found to be depleted 24 hpi. (C) Dorsal view of 1–4 days old male flies infected with 50 nl of 10-fold dilutions of 3×10^6^ TCID_50_ CrPV at day 0 and examined at 1, 2 and 3 dpi. The rectangle in the picture of a whole fly at the lower right corner indicates the approximate area shown in all other panels. (D) Quantitation of hemocyte numbers in CrPV-infected flies. Hemocyte depletion was estimated by counting the number of GFP spots visible under the dorsal cuticle of the first three abdominal segments. The results are representative of three independent experiments. (E) Survival of *Hml^Δ^-Gal4 UAS-GFP* males after injection of 50 nl of 10-fold dilutions of 3×10^6^ TCID_50_ CrPV. Note that hemocyte depletion precedes fly death. Bars represent mean values with standard error. (p<0.0001, Log-rank test for trend).

To further explore the role of hemocytes in antiviral responses, we next determined whether their localization changed during CrPV infection. With limited knowledge of CrPV's tissue tropism, we searched for any stereotypical pattern of localization that might suggest hemocyte homing. Hemocytes were labeled with GFP by using a *Hemolectin^Δ^*-Gal4 construct capable of driving expression of GFP specifically in hemocytes [46]. Since hemocytes are adherent and can be visualized through the cuticle by fluorescence microscopy, we followed their localization *in vivo* after CrPV injection. Because CrPV stocks were prepared from the supernatant of infected S2 cells, we ruled out non-specific effects by using the supernatant of mock-infected S2 cells as a negative control. PBS was also used as a negative control with similar results (data not shown). Though hemocytes concentrate at the injection site in the first hours after CrPV infection (Figure 3A), we did not observe any other stereotypical pattern of localization afterwards. Unexpectedly, we found that hemocytes were depleted in the course of CrPV infection in both larvae (Figure 3B) and adult flies (Figure 3C). A dose response curve further showed that the rate at which hemocytes were depleted was directly proportional to the dose of CrPV injected (Figure 3C–D) and that complete hemocyte depletion immediately preceded fly death (Figure 3D). Though we have not directly determined whether CrPV replicates in hemocytes *in vivo*, the strong cytopathic effects caused by CrPV infection of the hemocyte-like S2 cells [47] suggest that CrPV replication could lead to hemocyte death.

### Imd Pathway mutants are sensitive to CrPV infection

**Figure 4:**
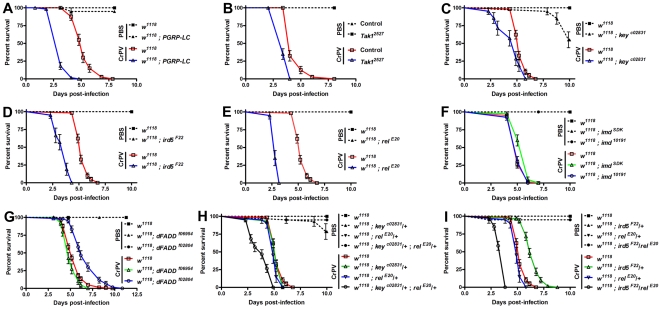
Imd pathway mutants are sensitive to CrPV infection. Homozygous (A–G) and transheterozygous (H–J) isogenic Imd pathway mutant males aged 1–4 days old were injected with 3×10^4^ TCID_50_ CrPV and incubated at 25°C while survival was monitored daily. (A) *PGRP-LC*, (B) *Tak1*, (C) *ird5*, (D) *kenny*, (E) *relish*, (F) *imd*, (G) *dFADD*, (H) *kenny*/+; *relish*/+, and (I) *ird5*/+; *relish*/+. All mutations (except *Tak1*, see methods) were maintained in an isogenic background after the crosses described in the Figure S1. Bars represent mean values with standard error.

**Figure 5:**
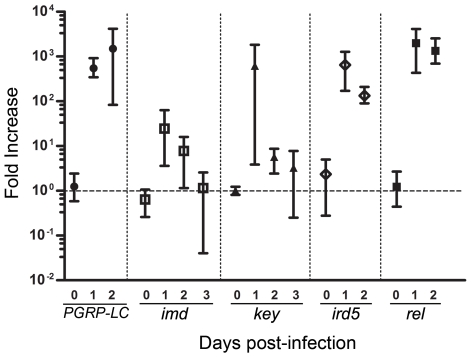
CrPV loads are increased in Imd pathway mutants. Viral RNA levels in Imd pathway mutants were measured by quantitative RT-PCR. Twenty 1–4 days old males were injected intra-abdominally on day 0 with 3×10_4_ TCID_50_ CrPV and incubated at 25°C. Groups of five flies were randomly selected and frozen immediately after injection (0) and after 1, 2, and 3 dpi (days post infection). To reduce experimental imprecision, only flies from vials in which all flies were still alive were analyzed. Bars represent mean values with experimental range from three independent experiments.

The evolutionarily conserved Toll and Imd signaling pathways are important signaling pathways in the fly. The Imd pathway is similar to the TNFR pathway, a major component of antiviral immunity in mammals. Preliminary results showed that homozygous Imd pathway mutants displayed an altered sensitivity to CrPV infection (data not shown). However, these mutations were in diverse genetic backgrounds (Figure 1B) and it could be argued that the phenotype was caused by background effects. This is supported by evidence that natural genetic variation considerably affects resistance to bacterial infection [48] and transmission of Sigma virus [49]. Since some genes of interest were obtained from the isogenic Exelixis collection [50], we chose to isogenize the genetic background of Imd pathway mutants by outcrossing them against the same isogenic Exelixis background strain (*w^1118^*) using the crossing scheme described in S1. To confirm that newly isogenized stocks retained the original Imd pathway mutations, we exploited the sensitivity of Imd pathway mutants to infections with Gram-negative bacteria and injected *Salmonella typhimurium* into homozygous mutants. All but control *w^1118^* flies were dead by 24 hpi (p<0.0001, data not shown). Next, isogenic homozygous mutants were infected with CrPV and their survival compared daily to control *w^1118^* flies. As shown in Figure 4A–E, homozygous mutations in *PGRP-LC*, *Tak1*, *ird5*, *key*, and *rel* were consistently more susceptible to CrPV challenge than control flies. Because these stocks were generated using a crossing scheme designed to isogenize all but the chromosome carrying the mutation to be tested (Figure S1), it remained possible that the phenotype was caused by homozygous background mutations on the same chromosome. To limit this possibility, we also tested double heterozygous combinations of Imd pathway mutations. Unlike homozygous combinations, which strongly disrupt or knock out one step in the pathway, double heterozygous combinations only reduce Imd signaling at two different points in the pathway. Therefore, a synergistic interaction between these mutations would be expected only if the Imd pathway was involved in antiviral responses. Indeed, double heterozygous combinations (+/*key^c02831^*; +/*rel^E20^* and i*rd5^F22^*/*rel^E20^*) displayed increased sensitivity to CrPV infection while single heterozygous controls were indistinguishable from background *w^1118^* flies (Figure 4H–I), except for heterozygous *ir5^F22^*/+ flies which developed resistance to CrPV infection (Figure 4I). To establish whether the increased sensitivity to CrPV infection was caused by a change in resistance or tolerance, we measured the viral loads in infected flies. We used qRT-PCR to determine CrPV loads in homozygous mutants at 1, 2, and 3 dpi. As shown in Figure 5, all mutants contained significantly higher CrPV loads at 1 and 2 dpi compared to *w^1118^* controls. Furthermore, those mutants most sensitive to CrPV infection (e.g., *PGRP-LC*, *ird5^F22^*, and *rel^E20^*) carried the highest viral loads. Altogether, these results indicate that the Imd pathway is required to mount an effective antiviral immune response against CrPV.

Unexpectedly, flies homozygous for either of two genes in the Imd pathway, *imd* (*imd^10191^*) and *dFADD* (*dFADD^f06954^*), did not display increased sensitivity to CrPV infection (Figure 4F–G). qRT-PCR analysis showed that viral loads were higher in *imd^10191^* compared to control flies at 1dpi, albeit not as high as shown by other Imd pathway mutants (Figure 5). However, CrPV loads dropped to control levels by 3 dpi suggesting a delayed but effective immune response was mounted against CrPV in *imd* mutants (Figure 5). A second *imd* allele (*imd^SDK^*) also behaved like control *w^1118^* flies while another allele of *dFADD* (*dFADD^f02804^*) conferred resistance to CrPV (Figure 4G, p<0.0001). Imd functions as an adaptor protein and transduces the signal from PGRP-LC, and possibly cytoplasmic PGRP-LE, downstream into two genetically distinct arms of the Imd pathway that converge in the activation of Rel. One arm leads to phosphorylation of Rel via Tak1-Key-Ird5 whereas the second arm directs cleavage of phosphorylated Rel through activation of the caspase Dredd via dFADD. Thus, *imd* is located at a branch-point in the Imd pathway while *dFADD* is located on the second branch of the pathway separated from the Tak1-Key-Ird5 branch. Recent evidence suggests that PGRP-LC can regulate and integrate different immune responses to infection [51] and that the interaction between PGRP-LC and Imd is not required to initiate signaling [14]. Taken together, these data indicate that Imd signaling is involved in antiviral responses against CrPV. Our results also suggest that the *imd* gene itself is dispensable for the response against CrPV and that distinct branches of the Imd pathway may contribute differently to antiviral immunity.

It was recently shown that flies infected with the Gram-negative bacteria *Wolbachia pipientis* are more resistant to infection with RNA viruses [52], including CrPV [43]. Because *Drosophila* is commonly infected with *Wolbachia*, it was possible that the differences in susceptibility to CrPV infection observed in the fly strains used in this study could be explained by the presence or absence of *Wolbachia*. Using PCR amplification of *wsp* gene sequences [53] we determined that all strains were infected with *Wolbachia* (data not shown). This indicates that the differences in sensitivity to CrPV infection observed in distinct wild type background strains (Figure 1B) as well as between Imd pathway mutants and *w^1118^* controls cannot be described simply by their *Wolbachia* status. These results conclusively show that Imd signaling is involved in antiviral responses in *Drosophila*.

## Discussion

We developed a genetically tractable *in vivo* host-virus model system by infecting *Drosophila* with CrPV. Because CrPV is a *Drosophila* pathogen, this offers a unique opportunity to study innate antiviral immunity in a natural system. To characterize the infection of CrPV in flies, rule out any potential artifacts, and optimize the experimental conditions we tested an array of biological variables. The lethal phenotype caused by injection of young *w^1118^* males with 3×10^4^ TCID_50_ at 25°C was robust and sensitive enough to be used an assay for the identification of mutations which confer increased susceptibility or resistance to CrPV infection.

To characterize the immune response to CrPV in the fly we investigated both humoral and cellular components of the immune system. A commonly studied aspect of the humoral response in the fly is the fast transcriptional activation of AMP genes following infection. Previous work has shown that infection of *Drosophila* with DXV induces AMP genes whose transcription is controlled by both Toll and Imd pathways. In contrast, Sigma Virus (SIGMAV) [29] and DCV [27] infection of *Drosophila* only weakly induced transcription of AMP genes and no AMPs were detected in the hemolymph of DCV-infected flies [54]. DCV and CrPV are closely related members of the *Dicistroviridae* family of non-enveloped positive ssRNA viruses. DXV belongs to the *Birnaviridae* family of non-enveloped bisegmented dsRNA viruses [55] whereas SIGMAV is an enveloped negative ssRNA Rhabdovirus that diverges from the other three viruses in several aspects of its life cycle [29]. Our results are consistent with the similarities between CrPV and DCV and indicate that infection of *Drosophila* with Dicistroviruses does not induce AMPs. The lack of AMP gene induction in SIGMAV-infected flies [29] and the lack of a protective effect in DXV-infected flies overexpressing single AMPs [56] suggest that the AMP response is not important for the immune response against viruses in *Drosophila*. It remains possible, however, that AMPs play a role during antiviral responses, but are actively suppressed by the virus. The induction of AMP genes observed in flies infected with DXV [56] may result from unique properties of DXV or differences in the experimental conditions.

Hemocytes form the cellular branch of the fly immune system and are involved in phagocytosis, melanization, and encapsulation of invading organisms [57] as well as signaling to activate the humoral response [58], [59]. The importance of hemocytes in fighting infections is evidenced by mutants lacking hemocytes [60], [61] or when phagocytosis is blocked by injection of polystyrene beads [21]. While mutations that reduce hemocyte numbers are lethal in adults [60], [61], bead injection provides a simple method to inhibit phagocytosis in adult hemocytes. Our results showed that phagocytosis-inhibited flies succumbed to a CrPV challenge faster than controls indicating that hemocyte-mediated phagocytosis is an important antiviral mechanism. Hemocytes have been shown to recognize virus-infected cells in Lepidoptera [62]. We investigated whether this was also the case in flies by tracking GFP-labeled hemocytes during the course of CrPV infection. Hemocytes were found to aggregate around the injection wound in the first 24 h after infection, but no other patterns were detected in the following days. However, we found that hemocytes throughout the fly were gradually depleted in a dose-dependent manner in CrPV-infected flies. Though we have not directly determined whether CrPV replicates in hemocytes *in vivo*, the strong cytopathic effects caused by CrPV infection of the hemocyte-like S2 cells [47] suggests that CrPV infection may cause hemocyte death.

To determine whether CrPV infection of *Drosophila* was a viable system for the identification of novel antiviral immunity genes, we took a candidate approach and asked whether conserved immune signaling pathways were involved in antiviral immunity. Since there is already evidence implicating Toll signaling in the immune response against DXV [56] in flies and Dengue virus in mosquitoes [25] we chose to test whether the Imd pathway was involved in antiviral responses. The Imd pathway is similar to the mammalian TNFR pathway, which plays a critical role in the immune response against viral infections. TNFR signaling interferes with viral replication by modulating the life cycle of infected cells or by directly affecting the viral life cycle [6]. Virtually all aspects of TNFR signaling have been shown to be targeted by viruses in an attempt to escape immune surveillance [63]. In flies, Imd signaling controls the expression of a set of AMP and other immunity genes in response to bacterial infections, but its role in antiviral immunity has not been thoroughly investigated. In this report, we examined whether the Imd pathway is required in the immune response against viral infections.

Consistent with the work of others [48], [49], our initial characterization of CrPV infection in flies showed that susceptibility to infection varies in different genetic backgrounds. Thus, to reduce confounding phenotypes arising from genetic background differences, we first isogenized all Imd pathway mutants of interest. CrPV infection of isogenic stocks showed that mutations in all but two Imd pathway genes increased the susceptibility to CrPV infection and led to higher CrPV loads when compared to controls. This clearly shows that the Imd pathway is required to mount an effective antiviral response against CrPV. Activation of the Imd pathway could be mediated by loss of PGRP-LC structural integrity in response to CrPV infection and tissue damage [51]. It is worth noting that *rel^E20^* flies, which carry a null mutation in the NF-κB transcription factor whose activation is controlled by Imd signaling, have been shown to be resistant to DCV infection [36], and lack any phenotype after DXV infection [56]. These conflicting results could be explained by variations in the genetic background and experimental conditions or result from differences in the properties of these viruses.

Our results also highlight the limitations of using AMP expression as the sole indicator for activation of immune pathways during infection. While it is well established that transcription of AMP genes depends upon the activation of Toll and Imd pathways, activation of these pathways is not always transduced in the induction of AMP genes. For example, we and others have shown that pathogen growth and, by extension, AMP transcription, can be uncoupled from survival [10], [56], [64], [65], [66], [67]. Thus, by using survival of mutant flies as a read out for Imd signaling we show that activation of the Imd pathway can be uncoupled from induction of AMP genes during antiviral responses. Activation of Imd pathway leads to transcription of several other genes; many of them with unknown functions and which could be involved in the regulation and execution of the immune response [7]. Alternatively, most of what we know about Imd signaling involves the fat body and it is possible that signaling affects antiviral immunity in another tissue. Our work suggests that hemocytes play an important role in fighting CrPV infection; gene activation in hemocytes is difficult to detect when assaying whole flies because hemocytes make up such a small proportion of the relative mass of the fly. Even if AMPs were induced in infected hemocytes as a result of Imd signaling, it would be difficult to see this change.

Two lines of evidence suggest that the two functionally distinct branches of the Imd signaling pathway contribute differently to antiviral immunity. First, AMP genes whose expression is controlled by Imd signaling are not induced during CrPV infection. Second, homozygous loss-of-function mutations in *imd* and *dFADD* were not susceptible to CrPV infection. The possibility that the two branches of the Imd pathway have distinct but overlapping functions is not unprecedented. Overexpression of Imd has been shown to induce apoptosis and constitutive AMP expression suggesting that immunity and apoptosis may share common control elements [68]. Apoptosis induced by Imd overexpression is suppressed in *Dredd* mutants but only slightly reduced in *dTAK1* mutants [20], indicating that apoptosis is mediated through the *dFADD-Dredd* branch but not the *dTAK1-kenny-Ird5* branch of the Imd pathway. It remains to be shown whether normal susceptibility to CrPV infection in *imd* and *dFADD* mutants is related to modulation of apoptosis insofar as a link between DCV and apoptosis has been suggested by induction of the caspase *Damm* in infected *Drosophila* [27] and several viruses have been shown to increase their fitness by blocking FADD-mediated apoptosis [6].

It was recently shown that the presence of *Wolbachia* in flies can change their susceptibility to CrPV infection [43]. This raises the possibility that differences in the immune response to CrPV in Imd pathway mutants could be explained by their *Wolbachia* status. We addressed this issue by showing that all flies in this study were infected with *Wolbachia*. However, it remains possible that different levels of *Wolbachia* in these mutants could influence, directly or indirectly, their susceptibility to CrPV infection.

## Methods

### Fly Stocks

Flies were maintained on standard molasses medium at 25°C. All experiments were performed with 1–4 days old males. To limit background effects, flies carrying mutations on the second and third chromosomes were isogenized to the *w^1118^* background following the schemes depicted on the Figure S1. Hemizygous *Tak1* males were obtained by crossing *Tak1* females to *w^1118^* males and compared to control flies generated by crossing *Tak1* males to *w^1118^* females.

The following fly stocks were obtained from the Bloomington stock center (http://fly.bio.indiana.edu/): *w^1118^* (Bloomington stock #6326), *imd^SDK^, kenny^c02831^*, and *rel^E20^*. PGRP-LC (*ird7*) was a generous gift of L. Wu, *ird5^F22^* was a gift of C. Brennan, *Tak1^2527^* was provided by M. Dionne, *imd^10191^* was previously described [9], *dFADD^f02804^* and *dFADD^f06954^* were obtained from the Exelixis collection maintained at the Harvard Medical School (https://Drosophila.med.harvard.edu/), and the *Hml^Δ^-Gal4 UAS-GFP* was described elsewhere [46].

### Virus titration

CrPV titration was performed according to Scotti (1977). Briefly, *Drosophila* Schneider Line-2 cells [69] were grown in Schneider's complete medium [70] supplemented with 10% fetal bovine serum (FBS) and antibiotics at a final concentration of 50 units Penicillin G and 50 µg Streptomycin Sulfate per ml of medium. CrPV suspensions were prepared as described before [38] and dilutions were prepared in complete Schneider's medium containing 10% FBS. For 50% Tissue Culture Infective Dose (TCID_50_) titrations, 96-well 3072 Microtest tissue culture plates (Becton Dickinson) were seeded with 1.5×10^6^ cells per well. The cells were then inoculated with 5 µl of serial dilutions of a CrPV suspension and incubated at 28°C. Cells were scored daily for 5 days for cytopathic effects (c.p.e.). the TCID_50_ was calculated using the Reed-Muench method [71]. The infectivity of the CrPV stock was determined to be 6×10^10^ TCID_50_/ml. In most experiments, flies were injected with 50 nl of a 1∶100 dilution in PBS which contained 3×10^4^ TCID_50_ CrPV.

### Injection assays

For CrPV and PBS injections, flies and third instar larvae (Figure 3B) were anesthetized with CO_2_ and injected with a total volume of 50 nl through a pulled glass needle. The injection volume was regulated using a Picospritzer III injector (Parker Hannifin, Rohnert Park, California, USA). The needle was introduced into the abdomen adult flies through the dorso-anterior surface adjacent to the hinge between the thorax and the abdomen. 1 to 4-day-old male flies were used for all experiments. After each injection, all flies were transferred to a new vial and maintained at 25°C. All experiments were performed in triplicates with twenty flies in each replicate. Each experiment was repeated at least three times. *w^1118^* flies were used as background strain.

*E. coli* DH5α cultures were grown overnight in Luria Broth (LB) at 37°C on a shaker and diluted to an OD_600_ of 0.1 just before injections.

### Quantitative RT-PCR

Triplicates of five flies were anesthetized, placed in 1.5-ml microfuge tubes, immediately frozen in dry ice, and stored at −80°C until RNA extraction. For RNA extraction flies were homogenized in 1 ml of Trizol-LS (Invitrogen, Cat #10296-010) with a plastic pestle, the homogenate was treated according to manufacturer's instructions, and RNA was resuspended in 30 µl of nuclease-free water. The remaining genomic DNA was degraded by treatment with RQ1 RNase-Free DNase (Promega Corporation, Madison, USA). RNA was then diluted 1∶1000 for RT-PCR reactions which were carried out in a Bio-Rad iCycler using a QuantiTect SYBR Green RT-PCR kit (Qiagen, Cat #204243) as directed by the manufacturer. The following primers were used to determine CrPV loads in flies: CGCACATTGACAGATGATAC (forward) and GTCTTCACATCTCCTGAACC (reverse). AMP transcript levels were determined using the following primers. Defensin: TTCTCGTGGCTATCGCTTTT (forward), GGAGAGTAGGTCGCATGTGG (reverse). Diptericin: ACCGCAGTACCCACTCAATC (forward), CCCAAGTGCTGTCCATATCC (reverse). Drosomycin: GTACTTGTTCGCCCTCTTCG (forward), CTTGCACACACGACGACAG (reverse). Relative RNA quantities were normalized to the *Drosophila* ribosomal protein 15a transcript in each sample using TGGACCACGAGGAGGCTAGG (forward) and GTTGGTTGCATGGTCGGTGA (reverse) primers.

### Bead Injections

Carboxylate-modified blue fluorescent 0.2-um diameter polystyrene beads (Molecular Probes) were injected to block phagocytosis as previously described [21]. Briefly, beads were washed three times in sterile water and resuspended in one fourth of the original volume in sterile water. Flies were injected with 50 nl of concentrated bead solution or water as an injection control. Three days later, the same flies were injected with CrPV or PBS as a negative control. For survival analysis, triplicates of 20 flies were injected for each condition, survival was scored daily, and data analyzed using GraphPad Prism (GraphPad Software). Flies were observed under a Leica MZ3 fluorescent dissecting microscope (Leica, Wetzlar, Germany) using GFP epifluorescence optics, and photographed with an ORCA camera (Hamamatsu, Osaka, Japan) using Openlab software (Improvision, Coventry, UK).

## Supporting Information

Figure S1 Genetic crosses used to isogenize Imd pathway mutants on the (A) second chromosome and (B) third chromosome. (6326) stands for chromosomes derived from the isogenic w1118; +; +stock (Bloomington stock number: 6326).
(8.32 MB TIF)

## References

[1] Ferrandon D, Imler JL, Hetru C, Hoffmann JA (2007). The Drosophila systemic immune response: sensing and signalling during bacterial and fungal infections.. Nat Rev Immunol.

[2] Rosetto M, Engstrom Y, Baldari CT, Telford JL, Hultmark D (1995). Signals from the IL-1 receptor homolog, Toll, can activate an immune response in a Drosophila hemocyte cell line.. Biochem Biophys Res Commun.

[3] Lemaitre B, Nicolas E, Michaut L, Reichhart JM, Hoffmann JA (1996). The dorsoventral regulatory gene cassette spatzle/Toll/cactus controls the potent antifungal response in Drosophila adults.. Cell.

[4] Medzhitov R, Preston-Hurlburt P, Janeway CA (1997). A human homologue of the Drosophila Toll protein signals activation of adaptive immunity.. Nature.

[5] Leulier F, Rodriguez A, Khush RS, Abrams JM, Lemaitre B (2000). The Drosophila caspase Dredd is required to resist gram-negative bacterial infection.. EMBO Rep.

[6] Herbein G, O'Brien WA (2000). Tumor necrosis factor (TNF)-alpha and TNF receptors in viral pathogenesis.. Proc Soc Exp Biol Med.

[7] De Gregorio E, Spellman PT, Tzou P, Rubin GM, Lemaitre B (2002). The Toll and Imd pathways are the major regulators of the immune response in Drosophila.. Embo J.

[8] Hedengren-Olcott M, Olcott MC, Mooney DT, Ekengren S, Geller BL (2004). Differential activation of the NF-kappaB-like factors Relish and Dif in Drosophila melanogaster by fungi and Gram-positive bacteria.. J Biol Chem.

[9] Pham LN, Dionne MS, Shirasu-Hiza M, Schneider DS (2007). A specific primed immune response in Drosophila is dependent on phagocytes.. PLoS Pathog.

[10] Dionne MS, Schneider DS (2008). Models of infectious diseases in the fruit fly *Drosophila melanogaster*.. Disease Models & Mechanisms.

[11] Choe KM, Werner T, Stoven S, Hultmark D, Anderson KV (2002). Requirement for a peptidoglycan recognition protein (PGRP) in Relish activation and antibacterial immune responses in Drosophila.. Science.

[12] Gottar M, Gobert V, Michel T, Belvin M, Duyk G (2002). The Drosophila immune response against Gram-negative bacteria is mediated by a peptidoglycan recognition protein.. Nature.

[13] Ramet M, Manfruelli P, Pearson A, Mathey-Prevot B, Ezekowitz RA (2002). Functional genomic analysis of phagocytosis and identification of a Drosophila receptor for E. coli.. Nature.

[14] Kaneko T, Yano T, Aggarwal K, Lim JH, Ueda K (2006). PGRP-LC and PGRP-LE have essential yet distinct functions in the drosophila immune response to monomeric DAP-type peptidoglycan.. Nat Immunol.

[15] Choe KM, Lee H, Anderson KV (2005). Drosophila peptidoglycan recognition protein LC (PGRP-LC) acts as a signal-transducing innate immune receptor.. Proc Natl Acad Sci U S A.

[16] Vidal S, Khush RS, Leulier F, Tzou P, Nakamura M (2001). Mutations in the Drosophila dTAK1 gene reveal a conserved function for MAPKKKs in the control of rel/NF-kappaB-dependent innate immune responses.. Genes Dev.

[17] Silverman N, Zhou R, Erlich RL, Hunter M, Bernstein E (2003). Immune activation of NF-kappaB and JNK requires Drosophila TAK1.. J Biol Chem.

[18] Rutschmann S, Jung AC, Zhou R, Silverman N, Hoffmann JA (2000). Role of Drosophila IKK gamma in a toll-independent antibacterial immune response.. Nat Immunol.

[19] Lu Y, Wu LP, Anderson KV (2001). The antibacterial arm of the drosophila innate immune response requires an IkappaB kinase.. Genes Dev.

[20] Leulier F, Vidal S, Saigo K, Ueda R, Lemaitre B (2002). Inducible expression of double-stranded RNA reveals a role for dFADD in the regulation of the antibacterial response in Drosophila adults.. Curr Biol.

[21] Elrod-Erickson M, Mishra S, Schneider D (2000). Interactions between the cellular and humoral immune responses in Drosophila.. Curr Biol.

[22] Stoven S, Silverman N, Junell A, Hedengren-Olcott M, Erturk D (2003). Caspase-mediated processing of the Drosophila NF-kappaB factor Relish.. Proc Natl Acad Sci U S A.

[23] Hedengren M, Asling B, Dushay MS, Ando I, Ekengren S (1999). Relish, a central factor in the control of humoral but not cellular immunity in Drosophila.. Mol Cell.

[24] Cherry S, Silverman N (2006). Host-pathogen interactions in drosophila: new tricks from an old friend.. Nat Immunol.

[25] Xi Z, Ramirez JL, Dimopoulos G (2008). The Aedes aegypti toll pathway controls dengue virus infection.. PLoS Pathog.

[26] Cherry S, Perrimon N (2004). Entry is a rate-limiting step for viral infection in a Drosophila melanogaster model of pathogenesis.. Nat Immunol.

[27] Dostert C, Jouanguy E, Irving P, Troxler L, Galiana-Arnoux D (2005). The Jak-STAT signaling pathway is required but not sufficient for the antiviral response of drosophila.. Nat Immunol.

[28] Hedges LM, Johnson KN (2008). Induction of host defence responses by Drosophila C virus.. J Gen Virol.

[29] Tsai CW, McGraw EA, Ammar ED, Dietzgen RG, Hogenhout SA (2008). Drosophila melanogaster mounts a unique immune response to the Rhabdovirus sigma virus.. Appl Environ Microbiol.

[30] Roxstrom-Lindquist K, Terenius O, Faye I (2004). Parasite-specific immune response in adult Drosophila melanogaster: a genomic study.. EMBO Rep.

[31] Zambon RA, Vakharia VN, Wu LP (2006). RNAi is an antiviral immune response against a dsRNA virus in Drosophila melanogaster.. Cell Microbiol.

[32] Chotkowski HL, Ciota AT, Jia Y, Puig-Basagoiti F, Kramer LD (2008). West Nile virus infection of Drosophila melanogaster induces a protective RNAi response.. Virology.

[33] Galiana-Arnoux D, Dostert C, Schneemann A, Hoffmann JA, Imler JL (2006). Essential function in vivo for Dicer-2 in host defense against RNA viruses in drosophila.. Nat Immunol.

[34] Wang XH, Aliyari R, Li WX, Li HW, Kim K (2006). RNA interference directs innate immunity against viruses in adult Drosophila.. Science.

[35] Saleh MC, Tassetto M, van Rij RP, Goic B, Gausson V (2009). Antiviral immunity in Drosophila requires systemic RNA interference spread.. Nature.

[36] Deddouche S, Matt N, Budd A, Mueller S, Kemp C (2008). The DExD/H-box helicase Dicer-2 mediates the induction of antiviral activity in drosophila.. Nat Immunol.

[37] Reinganun C, O'Loughlin GT, Hogan TW (1970). A nonoccluded virus of the field crickets *Teleogryllus oceanicus* and *T. commodus* (Orthoptera: Grylllidae).. J Invert Pathol.

[38] Scotti PD (1975). Cricket paralysis virus replicates in cultured Drosophila cells.. Intervirology.

[39] Reinganum C (1975). The isolation of cricket paralysis virus from the emperor gum moth, Antheraea eucalypti Scott, and its infectivity towards a range of insect species.. Intervirology.

[40] Fields BN, Knipe DM, Howley PM (2007). Fields' virology..

[41] Moore N, Kreuter J, Kurstak E (1991). Identification, Pathology, Structure, and Replication of Insect Picornaviruses.. Viruses of Invertebrates.

[42] Tate J, Liljas L, Scotti P, Christian P, Lin T (1999). The crystal structure of cricket paralysis virus: the first view of a new virus family.. Nat Struct Biol.

[43] Hedges LM, Brownlie JC, O'Neill SL, Johnson KN (2008). Wolbachia and virus protection in insects.. Science.

[44] van Rij RP, Saleh MC, Berry B, Foo C, Houk A (2006). The RNA silencing endonuclease Argonaute 2 mediates specific antiviral immunity in Drosophila melanogaster.. Genes Dev.

[45] Schneider D, Shahabuddin M (2000). Malaria parasite development in a Drosophila model.. Science.

[46] Sinenko SA, Mathey-Prevot B (2004). Increased expression of Drosophila tetraspanin, Tsp68C, suppresses the abnormal proliferation of ytr-deficient and Ras/Raf-activated hemocytes.. Oncogene.

[47] Scotti PD (1977). End-point dilution and plaque assay methods for titration of cricket paralysis virus in cultured Drosophila cells.. J Gen Virol.

[48] Lazzaro BP, Sceurman BK, Clark AG (2004). Genetic basis of natural variation in D. melanogaster antibacterial immunity.. Science.

[49] Bangham J, Kim KW, Webster CL, Jiggins FM (2008). Genetic variation affecting host-parasite interactions: different genes affect different aspects of sigma virus replication and transmission in Drosophila melanogaster.. Genetics.

[50] Thibault ST, Singer MA, Miyazaki WY, Milash B, Dompe NA (2004). A complementary transposon tool kit for Drosophila melanogaster using P and piggyBac.. Nat Genet.

[51] Schmidt RL, Trejo TR, Plummer TB, Platt JL, Tang AH (2008). Infection-induced proteolysis of PGRP-LC controls the IMD activation and melanization cascades in Drosophila.. FASEB J.

[52] Teixeira L, Ferreira A, Ashburner M (2008). The bacterial symbiont Wolbachia induces resistance to RNA viral infections in Drosophila melanogaster.. PLoS Biol.

[53] Zhou W, Rousset F, O'Neil S (1998). Phylogeny and PCR-based classification of Wolbachia strains using wsp gene sequences.. Proc Biol Sci.

[54] Sabatier L, Jouanguy E, Dostert C, Zachary D, Dimarcq JL (2003). Pherokine-2 and -3.. Eur J Biochem.

[55] Chung HK, Kordyban S, Cameron L, Dobos P (1996). Sequence analysis of the bicistronic Drosophila X virus genome segment A and its encoded polypeptides.. Virology.

[56] Zambon RA, Nandakumar M, Vakharia VN, Wu LP (2005). The Toll pathway is important for an antiviral response in Drosophila.. Proc Natl Acad Sci U S A.

[57] Lavine MD, Strand MR (2002). Insect hemocytes and their role in immunity.. Insect Biochem Mol Biol.

[58] Basset A, Khush RS, Braun A, Gardan L, Boccard F (2000). The phytopathogenic bacteria Erwinia carotovora infects Drosophila and activates an immune response.. Proc Natl Acad Sci U S A.

[59] Brennan CA, Delaney JR, Schneider DS, Anderson KV (2007). Psidin is required in Drosophila blood cells for both phagocytic degradation and immune activation of the fat body.. Curr Biol.

[60] Braun A, Hoffmann JA, Meister M (1998). Analysis of the Drosophila host defense in domino mutant larvae, which are devoid of hemocytes.. Proc Natl Acad Sci U S A.

[61] Braun A, Lemaitre B, Lanot R, Zachary D, Meister M (1997). Drosophila immunity: analysis of larval hemocytes by P-element-mediated enhancer trap.. Genetics.

[62] Trudeau D, Washburn JO, Volkman LE (2001). Central role of hemocytes in Autographa californica M nucleopolyhedrovirus pathogenesis in Heliothis virescens and Helicoverpa zea.. J Virol.

[63] Benedict CA, Banks TA, Ware CF (2003). Death and survival: viral regulation of TNF signaling pathways.. Curr Opin Immunol.

[64] Ayres JS, Freitag N, Schneider DS (2008). Identification of Drosophila mutants altering defense of and endurance to Listeria monocytogenes infection.. Genetics.

[65] Corby-Harris V, Habel KE, Ali FG, Promislow DE (2007). Alternative measures of response to Pseudomonas aeruginosa infection in Drosophila melanogaster.. J Evol Biol.

[66] Dionne MS, Pham LN, Shirasu-Hiza M, Schneider DS (2006). Akt and FOXO dysregulation contribute to infection-induced wasting in Drosophila.. Curr Biol.

[67] Schneider DS, Ayres JS, Brandt SM, Costa A, Dionne MS (2007). Drosophila eiger mutants are sensitive to extracellular pathogens.. PLoS Pathog.

[68] Georgel P, Naitza S, Kappler C, Ferrandon D, Zachary D (2001). Drosophila immune deficiency (IMD) is a death domain protein that activates antibacterial defense and can promote apoptosis.. Dev Cell.

[69] Schneider I (1972). Cell lines derived from late embryonic stages of Drosophila melanogaster.. J Embryol Exp Morphol.

[70] Schneider I (1964). Differentiation of Larval Drosophila Eye-Antennal Discs in Vitro.. J Exp Zool.

[71] Reed LJ, Muench H (1938). A simple method of estimating fifty percent endpoints.. Am J Hyg.

[72] Goto A, Kumagai T, Kumagai C, Hirose J, Narita H (2001). A Drosophila haemocyte-specific protein, hemolectin, similar to human von Willebrand factor.. Biochem J.

