# Supplementary figures and images for: The Imd Pathway Is Involved in Antiviral Immune Responses in Drosophila
Source: PLoS One. 2009 Oct 15;4(10):e7436. doi: 10.1371/journal.pone.0007436 (PMC2758544; doi:10.1371/journal.pone.0007436)

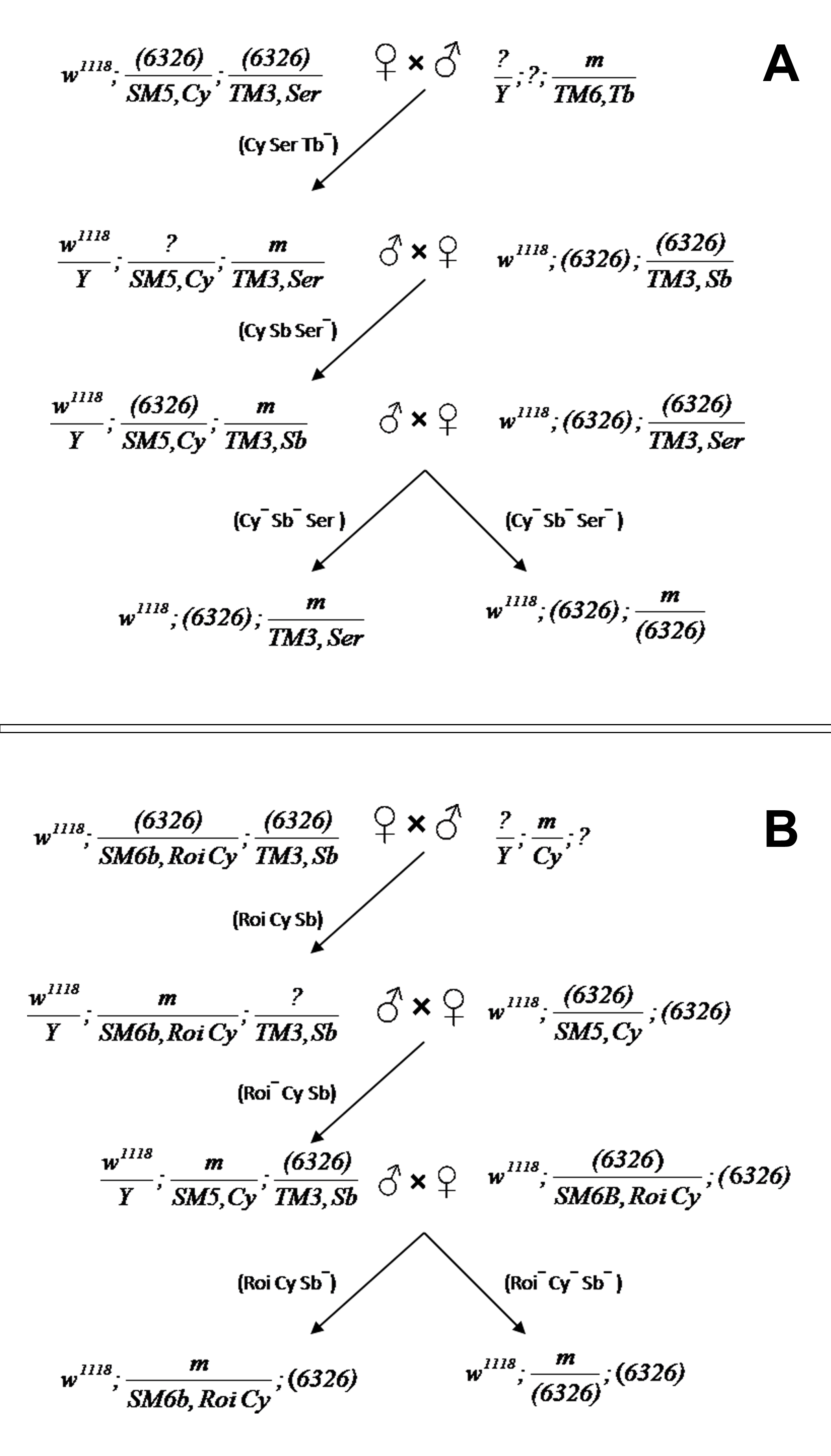

Supplement: Figure S1 — Genetic crosses used to isogenize Imd pathway mutants on the (A) second chromosome and (B) third chromosome. (6326) stands for chromosomes derived from the isogenic w1118; +; +stock (Bloomington stock number: 6326). (8.32 MB TIF) [file pone.0007436.s001.tif]
